# Supplementary material for: Pregnant Inuit Women’s Exposure to Metals and Association with Fetal Growth Outcomes: ACCEPT 2010–2015
Source: Int J Environ Res Public Health. 2019 Apr 1;16(7):1171. doi: 10.3390/ijerph16071171 (PMC6479494; doi:10.3390/ijerph16071171)
Supplement: Supplementary file 1 [file ijerph-16-01171-s001.zip › Table S1. Range and Function.docx]

**Table S1: Included metals, normal ranges and function**

| Heavy metals | Normal range (µg/L) [ref]  {Reference intake^1^} | Essential function^2^ |
| --- | --- | --- |
| Hg | 0.44-6.47 [1]; 0.46–7.5 [2] | - |
| Pb | 4-43 [1]; <50 [3] | - |
| As | <0.5-4.2 [2] | - |
| Cd | <0.030-0.317 [1] ; 0.09-0.54 [2] | - |
| Cr | <0.4-1.2 [1] ; <0.19-0.47 [2]  {40 µg} | Hormone activity, metabolism [4] |
| Mn | 7-18 [1]; 3.3-8.4 [2]  {2 mg} | Antioxidant, co-factor for enzymes, development, metabolism [5] |
| Ni | <0.3-0.77 [1] | Hormone activity, metabolism [6] |
| **Essential metals** |  |  |
| Se | 138-277 [1]; 75-125 [2]  {55 µg} | Antioxidant, immune system, co-factor for proteins and enzymes [7,8] |
| Fe ^3^ | 236-614 [1]; 390-550 [2]  {14 mg} | Oxygen transport, co-factor for proteins and enzymes [4] |
| Cu^3^ | 590-1470 [1]; 780-1760 [2]  {1 mg} | Growth, development, co-factor for enzymes [9] |
| Zn^3^ | 3500-9100 [1]; 3900-5700 [2]  {10 mg} | Antioxidant, immune system, co-factor for proteins and enzymes, wound healing [10,11] |
| Mg | ^†^  {375 mg} | Co-factor for enzymes and protein synthesis, metabolism [12] |
| Ca | ^†^  {800 mg} | Bone, cell function, hormones [13] |

^1^:Recommended daily intake as based on The Danish Veterinary and Food Administration under the Ministry of Environment and Food [14]; ^2^:Examples. Several more essential functions may exist. Applies to concentrations within the normal range.; ^3^:In mg/L; ^†^No reference available for whole blood.; ^[29]^ From a healthy non-pregnant, non-smoking, Swedish population 16-36 years of age, both male and female; ^[28]^ From a healthy Swedish population, both male and female; Variation depending on age, gender, physiological states (e.g., pregnancy), and season

1 Rodushkin I, Ödman F, Branth S. Multielement analysis of whole blood by high resolution inductively coupled plasma mass spectrometry. *Fresenius’ Journal of Analytical Chemistry* **1999**; 364(4):338–346. DOI: 10.1007/s002160051346.

2 Rodushkin I, Ödman F, Olofsson R, Burman E, Axelsson MD. Multi-element analysis of body fluids by double-focusing ICP-MS. *Recent Research Developments in Pure and Applied Chemistry* **2001**; 5(April 2001):51–66.

3 Centers for Disease Control and Prevention. Reference Blood Lead Levels (BLLs) for Adults in the U. S. Adult Blood Lead Epidemiol. Surveill. https://www.cdc.gov/niosh/topics/ables/description.html (accessed 7 May 2018).

4 Jaishankar M, Tseten T, Anbalagan N, Mathew BB, Beeregowda KN. Toxicity, mechanism and health effects of some heavy metals. *Interdisciplinary Toxicology* **2014**; 7(2):60–72. DOI: 10.2478/intox-2014-0009.

5 Avila DS, Puntel RL, Aschner M. Manganese in health and disease. *Metal ions in life sciences* **2013**; 13:199–227. DOI: 10.1007/978-94-007-7500-8_7.

6 Zdrojewicz Z, Popowicz E, Winiarski J. Nickel - role in human organism and toxic effects [in Polish]. *Polski merkuriusz lekarski : organ Polskiego Towarzystwa Lekarskiego* **2016**; 41(242):115–118.

7 Kurokawa S, Berry MJ. Selenium. Role of the Essential Metalloid in Health. *Metal ions in life sciences* **2013**; 13:499–534. DOI: 10.1007/978-94-007-7500-8_16.

8 Benstoem C, Goetzenich A, Kraemer S, Borosch S, Manzanares W, Hardy G, Stoppe C. Selenium and Its Supplementation in Cardiovascular Disease—What do We Know? *Nutrients* **2015**; 7(5):3094–3118. DOI: 10.3390/nu7053094.

9 World Health Organization. Adverse Health Effects of Heavy Metals in Children. *Children’s Health and the Environment WHO Training Package for the Health Sector World* **2011**; 1–77.

10 Chasapis CT, Loutsidou AC, Spiliopoulou CA, Stefanidou ME. Zinc and human health: an update. *Archives of toxicology* **2012**; 86(4):521–534. DOI: 10.1007/s00204-011-0775-1.

11 Lin P-H, Sermersheim M, Li H, Lee PHU, Steinberg SM, Ma J. Zinc in Wound Healing Modulation. *Nutrients* **2018**; 10(1). DOI: 10.3390/nu10010016 doi:10.3390/nu10010016.

12 de Baaij JHF, Hoenderop JGJ, Bindels RJM. Magnesium in man: implications for health and disease. *Physiological reviews* **2015**; 95(1):1–46. DOI: 10.1152/physrev.00012.2014.

13 Martinez de Victoria E. Calcium, essential for health. *Nutricion hospitalaria* **2016**; 33(Suppl 4):341.

14 Miljø- og Fødevareministeriet. Vejledning om næringsdeklaration. **2016**.
