# Supplementary material for: Pregnant Inuit Women’s Exposure to Metals and Association with Fetal Growth Outcomes: ACCEPT 2010–2015
Source: Int J Environ Res Public Health. 2019 Apr 1;16(7):1171. doi: 10.3390/ijerph16071171 (PMC6479494; doi:10.3390/ijerph16071171)
Supplement: Supplementary file 1 [file ijerph-16-01171-s001.zip › Table S14-S18. Regional Metal correlation.docx]

**Table S14** Spearmans correlation coefficient (r_s_) between identified metals for North region. N=33

|  | Hg | Se | Plasma-Se | Pb | As | Cd | Cr | Mn | Fe | Cu | Zn | Ni | Mg | Ca |
| --- | --- | --- | --- | --- | --- | --- | --- | --- | --- | --- | --- | --- | --- | --- |
| Hg |  |  |  |  |  |  |  |  |  |  |  |  |  |  |
| Se | **0.635** |  |  |  |  |  |  |  |  |  |  |  |  |  |
| Plasma-Se | **0.650** | **0.616** |  |  |  |  |  |  |  |  |  |  |  |  |
| Pb | 0.129 | **0.446** | 0.050 |  |  |  |  |  |  |  |  |  |  |  |
| As | -0.152 | -0.057 | -0.249 | **0.393** |  |  |  |  |  |  |  |  |  |  |
| Cd | 0.091 | -0.194 | -0.114 | 0.039 | -0.063 |  |  |  |  |  |  |  |  |  |
| Cr | -0.344 | -0.103 | **-0.369** | 0.262 | **0.785** | -0.179 |  |  |  |  |  |  |  |  |
| Mn | -0.161 | -0.153 | **-0.439** | 0.220 | 0.239 | 0.295 | 0.337 |  |  |  |  |  |  |  |
| Fe* | 0.287 | 0.175 | 0.274 | 0.265 | -0.020 | 0.245 | -0.054 | -0.046 |  |  |  |  |  |  |
| Cu | 0.050 | -0.027 | 0.090 | **-0.418** | **-0.407** | 0.216 | **-0.380** | 0.109 | -0.170 |  |  |  |  |  |
| Zn | 0.033 | -0.051 | -0.137 | 0.189 | 0.004 | 0.086 | 0.046 | **0.399** | **0.422** | 0.027 |  |  |  |  |
| Ni | -0.060 | -0.039 | 0.217 | -0.054 | 0.055 | -0.086 | -0.033 | **-0.525** | 0.326 | -0.229 | -0.279 |  |  |  |
| Mg* | **0.470** | 0.179 | 0.218 | 0.129 | 0.031 | 0.096 | -0.092 | -0.155 | 0.432 | 0.113 | 0.080 | 0.038 |  |  |
| Ca* | 0.160 | -0.035 | 0.356 | -0.203 | -0.226 | 0.225 | -0.266 | -0.172 | 0.005 | -0.018 | -0.173 | 0.189 | 0.220 |  |

r_s_-values in bold lists statistically significant correlation (p<0.05); *: Calculated with less data than for the rest of the metals

**Table S15.** Spearmans correlation coefficient (r_s_) between identified metals for Disko Bay region. N=123

|  | Hg | Se | Plasma-Se | Pb | As | Cd | Cr | Mn | Fe | Cu | Zn | Ni | Mg | Ca |
| --- | --- | --- | --- | --- | --- | --- | --- | --- | --- | --- | --- | --- | --- | --- |
| Hg |  |  |  |  |  |  |  |  |  |  |  |  |  |  |
| Se | **0.689** |  |  |  |  |  |  |  |  |  |  |  |  |  |
| Plasma-Se | **0.211** | **0.339** |  |  |  |  |  |  |  |  |  |  |  |  |
| Pb | **0.422** | **0.410** | **0.251** |  |  |  |  |  |  |  |  |  |  |  |
| As | **0.289** | **0.276** | 0.120 | **0.245** |  |  |  |  |  |  |  |  |  |  |
| Cd | 0.079 | -0.098 | **-0.281** | -0.048 | **-0.208** |  |  |  |  |  |  |  |  |  |
| Cr | 0.006 | 0.096 | 0.054 | 0.146 | **0.683** | **-0.275** |  |  |  |  |  |  |  |  |
| Mn | 0.037 | 0.023 | 0.062 | 0.008 | **0.552** | 0.108 | **0.723** |  |  |  |  |  |  |  |
| Fe* | 0.135 | 0.043 | 0.078 | 0.117 | 0.061 | **0.184** | **0.187** | 0.111 |  |  |  |  |  |  |
| Cu | 0.009 | -0.054 | -0.158 | **-0.228** | -0.128 | **0.341** | **-0.177** | 0.031 | -0.107 |  |  |  |  |  |
| Zn | 0.092 | 0.091 | 0.060 | 0.068 | -0.044 | -0.148 | 0.130 | 0.150 | **0.455** | -0.170 |  |  |  |  |
| Ni | -0.152 | -0.116 | -0.100 | 0.002 | **-0.391** | **0.245** | -0.128 | **-0.336** | **0.175** | 0.150 | 0.031 |  |  |  |
| Mg* | 0.143 | 0.173 | **0.371** | 0.134 | **0.285** | -0.113 | 0.421 | **0.413** | **0.632** | **-0.265** | **0.490** | **-0.353** |  |  |
| Ca* | -0.092 | 0.039 | 0.057 | -0.159 | **-0.226** | -0.075 | -0.305 | **-0.310** | **-0.334** | **0.397** | -0.196 | 0.198 | -0.150 |  |

r_s_-values in bold lists statistically significant correlation (p<0.05); *: Calculated with less data than for the rest of the metals

**Table S16.** Spearmans correlation coefficient (r_s_) between identified metals for West region. N=289

|  | Hg | Se | Plasma-Se | Pb | As | Cd | Cr | Mn | Fe | Cu | Zn | Ni | Mg | Ca |
| --- | --- | --- | --- | --- | --- | --- | --- | --- | --- | --- | --- | --- | --- | --- |
| Hg |  |  |  |  |  |  |  |  |  |  |  |  |  |  |
| Se | **0.395** |  |  |  |  |  |  |  |  |  |  |  |  |  |
| Plasma-Se | **0.121** | **0.323** |  |  |  |  |  |  |  |  |  |  |  |  |
| Pb | **0.135** | **0.297** | -0.010 |  |  |  |  |  |  |  |  |  |  |  |
| As | **0.236** | **0.303** | -0.028 | 0.063 |  |  |  |  |  |  |  |  |  |  |
| Cd | -0.078 | **-0.120** | -0.051 | -0.033 | -0.091 |  |  |  |  |  |  |  |  |  |
| Cr | -0.031 | 0.112 | **-0.205** | 0.076 | **0.525** | **-0.216** |  |  |  |  |  |  |  |  |
| Mn | 0.011 | 0.099 | **-0.197** | 0.116 | **0.491** | 0.033 | **0.612** |  |  |  |  |  |  |  |
| Fe | **0.183** | **0.261** | 0.045 | **0.133** | 0.031 | **0.191** | 0.111 | 0.124 |  |  |  |  |  |  |
| Cu* | 0.027 | -0.098 | 0.081 | **-0.154** | **-0.187** | **0.457** | **-0.267** | -0.081 | -0.062 |  |  |  |  |  |
| Zn | **0.124** | **0.143** | -0.010 | 0.055 | **0.126** | -0.048 | 0.101 | **0.178** | **0.424** | **-0.134** |  |  |  |  |
| Ni | -0.059 | -0.102 | 0.072 | -0.080 | **-0.415** | **0.132** | -0.075 | **-0.358** | **0.153** | **0.124** | -0.094 |  |  |  |
| Mg* | **0.225** | **0.293** | -0.009 | 0.137 | **0.261** | -0.119 | **0.237** | **0.263** | **0.457** | **-0.202** | **0.181** | **-0.263** |  |  |
| Ca* | **0.212** | 0.011 | **0.199** | **-0.130** | **-0.231** | **0.233** | **-0.312** | **-0.337** | **-0.144** | **0.519** | **-0.191** | **0.295** | -0.016 |  |

r_s_-values in bold lists statistically significant correlation (p<0.05); *: Calculated with less data than for the rest of the metals

**Table S17.** Spearmans correlation coefficient (r_s_) between identified metals for South region. N=44

|  | Hg | Se | Plasma-Se | Pb | As | Cd | Cr | Mn | Fe | Cu | Zn | Ni | Mg | Ca |
| --- | --- | --- | --- | --- | --- | --- | --- | --- | --- | --- | --- | --- | --- | --- |
| Hg |  |  |  |  |  |  |  |  |  |  |  |  |  |  |
| Se | **0.408** |  |  |  |  |  |  |  |  |  |  |  |  |  |
| Plasma-Se | 0.141 | 0.061 |  |  |  |  |  |  |  |  |  |  |  |  |
| Pb | 0.124 | **0.357** | 0.057 |  |  |  |  |  |  |  |  |  |  |  |
| As | -0.070 | 0.090 | -0.116 | 0.160 |  |  |  |  |  |  |  |  |  |  |
| Cd | 0.086 | -0.029 | -0.203 | 0.040 | 0.047 |  |  |  |  |  |  |  |  |  |
| Cr | **-0.397** | -0.109 | -0.269 | 0.113 | **0.529** | -0.134 |  |  |  |  |  |  |  |  |
| Mn | 0.079 | 0.187 | -0.247 | 0.272 | 0.618 | **0.345** | **0.521** |  |  |  |  |  |  |  |
| Fe | 0.228 | 0.325 | -0.029 | 0.391 | 0.133 | 0.162 | -0.030 | 0.233 |  |  |  |  |  |  |
| Cu* | -0.051 | -0.240 | **0.315** | -0.234 | **-0.298** | 0.060 | -0.124 | -0.009 | **-0.302** |  |  |  |  |  |
| Zn | 0.087 | 0.096 | -0.174 | **0.377** | 0.005 | 0.289 | 0.019 | 0.257 | **0.621** | **-0.300** |  |  |  |  |
| Ni | -0.103 | -0.026 | 0.106 | **-0.303** | -0.264 | **-0.236** | 0.042 | **-0.426** | -0.052 | -0.134 | -0.086 |  |  |  |
| Mg* | 0.035 | 0.342 | -0.228 | **0.549** | **0.510** | 0.068 | 0.336 | **0.573** | **0.668** | **-0.731** | **0.417** | **-0.523** |  |  |
| Ca* | -0.103 | **-0.418** | 0.223 | **-0.715** | **-0.465** | -0.037 | -0.275 | **-0.509** | **-0.655** | **0.757** | **-0.471** | 0.354 | **-0.844** |  |

r_s_-values in bold lists statistically significant correlation (p<0.05); *: Calculated with less data than for the rest of the metals

**Table S18.** Spearmans correlation coefficient (r_s_) between identified metals for East region. N=20

|  | Hg | Se | Plasma-Se | Pb | As | Cd | Cr | Mn | Fe | Cu | Zn | Ni | Mg | Ca |
| --- | --- | --- | --- | --- | --- | --- | --- | --- | --- | --- | --- | --- | --- | --- |
| Hg |  |  |  |  |  |  |  |  |  |  |  |  |  |  |
| Se | 0.096 |  |  |  |  |  |  |  |  |  |  |  |  |  |
| Plasma-Se | -0.108 | 0.226 |  |  |  |  |  |  |  |  |  |  |  |  |
| Pb | 0.094 | -0.015 | 0.020 |  |  |  |  |  |  |  |  |  |  |  |
| As | -0.250 | 0.406 | **0.448** | 0.049 |  |  |  |  |  |  |  |  |  |  |
| Cd | -0.090 | 0.400 | 0.085 | 0.052 | 0.301 |  |  |  |  |  |  |  |  |  |
| Cr | 0.032 | -0.057 | -0.221 | 0.432 | 0.093 | -0.163 |  |  |  |  |  |  |  |  |
| Mn | 0.072 | -0.164 | -0.054 | **0.537** | 0.274 | -0.104 | **0.640** |  |  |  |  |  |  |  |
| Fe | 0.188 | 0.111 | **0.493** | 0.230 | 0.291 | -0.091 | 0.090 | **0.453** |  |  |  |  |  |  |
| Cu* | -0.071 | -0.250 | -0.214 | -0.714 | -0.694 | 0.408 | -0.704 | **-0.821** | -0.179 |  |  |  |  |  |
| Zn | -0.140 | -0.047 | 0.073 | 0.158 | 0.166 | -0.077 | 0.219 | **0.639** | **0.702** | -0.336 |  |  |  |  |
| Ni | 0.239 | **-0.498** | -**0.507** | -0.167 | **-0.773** | **-0.543** | 0.048 | -0.194 | -0.287 | 0.058 | -0.207 |  |  |  |
| Mg* | 0.714 | -0.086 | 0.486 | 0.257 | 0.488 | 0.334 | 0.577 | 0.714 | **0.829** | -0.543 | **-0.829** | -0.488 |  |  |
| Ca* | -0.543 | -0.600 | -0.600 | -0.771 | -0.293 | 0.030 | -0.334 | -0.371 | -0.714 | 0.657 | -0.086 | 0.293 | -0.486 |  |

r_s_-values in bold lists statistically significant correlation (p<0.05); *: Calculated with less data than for the rest of the metals
