# Supplementary material for: Pregnant Inuit Women’s Exposure to Metals and Association with Fetal Growth Outcomes: ACCEPT 2010–2015
Source: Int J Environ Res Public Health. 2019 Apr 1;16(7):1171. doi: 10.3390/ijerph16071171 (PMC6479494; doi:10.3390/ijerph16071171)
Supplement: Supplementary file 1 [file ijerph-16-01171-s001.zip › Table S19. Measured ACCEPT Metals.docx]

**Table S19**. The measured metals in ACCEPT

| Sampling year 2010-2011  Test report no. 765(n=212)  Measure date July, 2014 | | | Sampling year 2013-2015  Test report no. 2015-845(n=178)  Measure date April, 2015 | | | Sampling year 2013-2015  Test report no. 2015-815(n=17)  Measure date April, 2015 | | | Sampling year 2013-2015 (new grant)  Test report no. 943(n=184)  Measure date Jan, 2018 | | |
| --- | --- | --- | --- | --- | --- | --- | --- | --- | --- | --- | --- |
| metal | **DL** | % over DL | metal | **DL** | % over DL | metal | **DL** | % over DL | metal | **DL** | % over DL |
| Mg WB | **0.4** | 100 | Mg WB | **0.9** | 100 | Mg WB# |  |  |  |  |  |
| Ca WB | **4.5** | 100 | Ca WB | **3.8** | 100 | Ca WB# |  |  |  |  |  |
| Cr WB | **0.013** | 15.4 | Cr WB | **0.024** | 100 | Cr WB | **0.025** | 0 | Cr WB | **0.032** | 20.3 |
| Mn WB | **0.006** | 97.1 | Mn WB | **0.002** | 100 | Mn WB | **0.01** | 100 | Mn WB | **0.007** | 87.3 |
| Fe WB | **2.315** | 100 | Fe WB | **0.2** | 100 | Fe WB | **0.5** | 100 | Fe WB | **0.47** | 100 |
| Ni WB | **0.023** | 4.83 | Ni WB | **0.011** | 3.06 | Ni WB | **0.034** | 0 | Ni WB | **0.043** | 22.3 |
| Cu WB | **0.027** | 100 | Cu WB | **0.017** | 100 | Cu WB# |  |  | Cu WB | **0.007** | 100 |
| Zn WB | **0.043** | 100 | Zn WB | **0.04** | 100 | Zn WB | **0.01** | 100 | Zn WB | **0.071** | 100 |
| As WB | **0.008** | 5.31 | As WB | **0.015** | 25.51 | As WB | **0.004** | 23.53 | As WB | **0.002** | 88.3 |
| Se WB | **0.031** | 100 | Se WB | **0.004** | 100 | Se WB | **0.012** | 100 | Se WB | **0.0004** | 100 |
| Se_plasma |  | 99.5 | Se_plasma | **0.028** | 100 | Se_plasma |  | 100 | Se_plasma | **0.0008** | 100 |
| Cd WB | **0.003** | 9.18 | Cd WB | **0.001** | 54.08 | Cd WB | **0.0028** | 0 | Cd WB | **0.0002** | 76.7 |
| Hg WB | **0.0016** | 84.54 | Hg WB | **0.004** | 48.98 | Hg WB | **0.0028** | 82.35 | Hg WB | **0.0007** | 97.5 |
| Pb WB | **0.008** | 39.13 | Pb WB | **0.003** | 97.96 | Pb WB | **0.0134** | 11.76 | Pb WB | **0.0025** | 100 |

#: 17 samples not measured; WB: whole blood; DL: detect limit (mg/kg)
