# Supplementary material for: Pregnant Inuit Women’s Exposure to Metals and Association with Fetal Growth Outcomes: ACCEPT 2010–2015
Source: Int J Environ Res Public Health. 2019 Apr 1;16(7):1171. doi: 10.3390/ijerph16071171 (PMC6479494; doi:10.3390/ijerph16071171)
Supplement: Supplementary file 1 [file ijerph-16-01171-s001.zip › Table S2. Spearmans Correlation All Regions.docx]

**Table S2**. Spearmans correlation coefficient (r_s_) between the blood metals including all regions^1^. n=502

| Metal | Hg | Se | Plasma-Se | Pb | As | Cd | Cr | Mn | Fe | Cu | Zn | Ni | Mg |
| --- | --- | --- | --- | --- | --- | --- | --- | --- | --- | --- | --- | --- | --- |
| Hg |  |  |  |  |  |  |  |  |  |  |  |  |  |
| Se | **0.481** |  |  |  |  |  |  |  |  |  |  |  |  |
| Plasma-Se | **0.160** | **0.319** |  |  |  |  |  |  |  |  |  |  |  |
| Pb | **0.212** | **0.338** | 0.071 |  |  |  |  |  |  |  |  |  |  |
| As | **0.154** | **0.256** | 0.032 | **0.113** |  |  |  |  |  |  |  |  |  |
| Cd | **0.103** | **-0.090** | **-0.119** | -0.018 | **-0.103** |  |  |  |  |  |  |  |  |
| Cr | -0.056 | 0.085 | **-0.162** | **0.098** | **0.568** | **-0.226** |  |  |  |  |  |  |  |
| Mn | 0.005 | 0.055 | **-0.154** | **0.130** | **0.507** | **0.064** | **0.610** |  |  |  |  |  |  |
| Fe | **0.118** | **0.177** | **0.099** | **0.115** | 0.064 | **0.138** | **0.098** | **0.112** |  |  |  |  |  |
| Cu^2^ | 0.019 | **-0.097** | 0.044 | **-0.194** | **-0.204** | **0.375** | **-0.277** | -0.067 | **-0.134** |  |  |  |  |
| Zn | 0.069 | 0.078 | -0.023 | 0.086 | 0.083 | -0.009 | **0.103** | **0.211** | **0.475** | **-0.141** |  |  |  |
| Ni | -0.016 | -0.077 | 0.065 | **-0.089** | **-0.419** | 0.043 | **-0.137** | **-0.423** | **0.102** | 0.062 | **-0.112** |  |  |
| Mg^2^ | **0.170** | **0.232** | 0.090 | **0.165** | **0.272** | -0.094 | **0.287** | **0.323** | **0.537** | **-0.238** | **0.304** | **-0.291** |  |
| Ca^2^ | 0.094 | -0.019 | **0.143** | **-0.195** | **-0.246** | **0.140** | **-0.304** | **-0.339** | **-0.257** | **0.483** | **-0.215** | **0.267** | **-0.124** |

r_s_-values: **bold** indicate statistically significant correlation (p<0.05). Positive correlations in **black** and negative correlations in **red**; ^1^: The region the women lived for >50% of their lives; ^2^: Calculated with less data: Cu n=488, Mg n=344 and Ca n=344
