# Supplementary material for: Pregnant Inuit Women’s Exposure to Metals and Association with Fetal Growth Outcomes: ACCEPT 2010–2015
Source: Int J Environ Res Public Health. 2019 Apr 1;16(7):1171. doi: 10.3390/ijerph16071171 (PMC6479494; doi:10.3390/ijerph16071171)
Supplement: Supplementary file 1 [file ijerph-16-01171-s001.zip › Table S20a-20b. Quartiles of metal concentration.docx]

**Table S20a.** Birth outcomes divided into quartiles of heavy metal concentration

|  | | Birth weight (grams) | | Birth length (cm) | | Head circumference (cm) | | Gestation age (week) | | APGAR 5 min | |
| --- | --- | --- | --- | --- | --- | --- | --- | --- | --- | --- | --- |
| Metal | Quartile | n | Mean (SD) | n | Mean (SD) | n | Mean (SD) | n | Mean (SD) | n | Mean (SD) |
| Hg | 1  2  3  4  p | 87  137  131  126 | 3551.1 (593.2)  3590.9 (523.1)  3621.6 (654.1)  3513.9 (543.3)  0.478 | 87  137  131  126 | 51.3 (3.0)  51.5 (2.2)  51.3 (3.5)  51.0 (2.8)  0.495 | 87  137  130  125 | 34.7 (1.6)  34.8 (1.5)  34.7 (1.9)  34.4 (1.9)  0.387 | 84  134  129  117 | 39.0 (1.6)  39.3 (1.6)  39.2 (2.2)  38.9 (2.2)  0.325 | 86  135  129  125 | 9.7 (0.9)  9.9 (0.5)  9.8 (1.1)  9.7 (1.0)  0.469 |
|  |  |  |  |  |  |  |  |  |  |  |  |
| Pb | 1  2  3  4  p | 159  89  109  116 | 3565.9 (525.5)  3683.0 (553.1)  3643.1 (564.8)  3433.4 (580.9)  **0.009** | 159  89  109  116 | 51.3 (2.5)  51.4 (3.8)  51.6 (2.5)  50.8 (3.1)  0.145 | 158  89  109  115 | 34.7(1.5)  34.9 (1.9)  34.7 (1.6)  34.3 (2.0)  **0.049** | 151  88  104  113 | 39.0 (1.6)  39.3 (2.0)  39.4 (1.8)  38.9 (2.3)  0.249 | 155  89  108  115 | 9.9 (0.5)  9.6 (1.1)  9.7 (1.1)  9.8 (0.9)  0.178 |
|  |  |  |  |  |  |  |  |  |  |  |  |
| As | 1  2  3  4  p | 66  186  156  65 | 3658.8 (504.8)  3490.9 (551.1)  3632.8 (638.6)  3579.1 (573.3)  0.105 | 66  186  156  65 | 51.7 (2.8)  51.2 (2.4)  51.1 (3.7)  51.3 (2.4)  0.635 | 66  185  155  65 | 34.8 (1.4)  35.6 (1.5)  34.7 (2.1)  34.4 (1.7)  0.490 | 63  179  151  63 | 39.9 (1.3)  38.9 (1.6)  39.0 (2.4)  39.2 (1.8)  **0.006** | 64  183  155  65 | 9.9 (0.3)  9.9 (0.5)  9.6 (1.4)  9.8 (0.6)  0.069 |
|  |  |  |  |  |  |  |  |  |  |  |  |
| Cd | 1  2  3  4  p | 77  140  202  54 | 3764.2 (555.5)  3595.6 (662.1)  3518.0 (524.7)  3449.4 (536.4)  **0.005** | 92  158  202  56 | 52.0 (2.8)  51.0 (3.8)  51.3 (2.3)  50.7 (2.3)  0.054 | 77  140  202  52 | 35.0 (1.8)  34. 6 (2.1)  34.6 (1.5)  34.4 (1.4)  0.198 | 76  136  193  51 | 39.7 (1.6)  39.0 (2.5)  39.0 (1.7)  38.9 (1.4)  **0.027** | 75  139  199  54 | 9.7 (0.9)  9.7 (1.3)  9.9 (0.5)  10.0 (0.3)  0.074 |
|  |  |  |  |  |  |  |  |  |  |  |  |
| Cr | 1  2  3  4  p | 199  26  140  108 | 3543.1 (547.4)  3644.0 (466.1)  3596.8 (554.3)  3581.3 (693.4)  0.761 | 199  26  140  108 | 51.4 (2.4)  51.6 (2.0)  51.4 (2.5)  50.9 (4.2)  0.594 | 199  26  138  108 | 34. 7 (1.5)  34.7 (1.0)  34.6 (1.6)  34.6 (2.3)  0.959 | 193  23  137  103 | 39.1 (1.6)  39.8 (1.6)  39.2 (1.6)  38.9 (2.8)  0.245 | 196  26  138  107 | 9.9 (0.5)  9.9 (0.2)  9.8 (0.7)  9.6 (1.5)  **0.024** |
|  |  |  |  |  |  |  |  |  |  |  |  |
| Mn | 1  2  3  4  p | 116  127  114  116 | 3596.0 (595.9)  3521.3 (610.6)  3509.2 (565.2)  3670.2 (565.2)  0.121 | 116  127  114  116 | 51.2 (3.1)  51.2 (2.8)  51.2 (2.4)  51.4 (3.4)  0.937 | 115  126  114  116 | 34.6 (1.9)  34.6 (1.8)  34.4 (1.4)  34.9 (1.8)  0.168 | 116  121  108  111 | 39.1 (2.2)  39.2 (1.8)  39.0 (1.7)  39.2 (2.0)  0.846 | 114  124  114  115 | 9.7 (1.1)  9.8 (0.6)  9.8 (0.6)  9.8 (1.1)  0.774 |
|  |  |  |  |  |  |  |  |  |  |  |  |
| Ni | 1  2  3  4  p | 161  10  187  115 | 3660.1 (566.2)  3732.9 (358.8)  3502.8 (558.3)  3552.3 (638.0)  0.064 | 161  10  187  115 | 51.4 (3.2)  51.6 (1.8)  51.3 (2.4)  51.1 (3.4)  0.917 | 161  10  186  114 | 34.8 (1.7)  34.9 (0.9)  34.6 (1.5)  34.5 (2.1)  0.378 | 156  10  180  110 | 39.3 (2.0)  40.3 (0.7)  38.9 (1.6)  39.1 (1.9)  0.074 | 160  10  184  113 | 9.8 (0.8)  10.0 (0.0)  9.9 (0.5)  9.6 (1.4)  0.088 |

p-value calculated by One-way ANOVA on crude non-adjusted data

**Table S20b.** Birth outcomes divided into quartiles of essential metal concentration

|  | | Birth weight (grams) | | Birth length (cm) | | Head circumference (cm) | | Gestation age (week) | | APGAR 5 min | |
| --- | --- | --- | --- | --- | --- | --- | --- | --- | --- | --- | --- |
| Metal | Quartile | n | Mean (SD) | n | Mean (SD) | n | Mean (SD) | n | Mean (SD) | n | Mean (SD) |
| Se | 1  2  3  4  p | 116  114  123  120 | 3534.6 (544.8)  3601.7 (534.6)  3598.5 (610.7)  3557.6 (628.4)  0.777 | 116  114  123  120 | 51.1 (2.5)  51.4 (2.4)  51.3 (3.4)  51.2 (3.1)  0.877 | 116  114  122  119 | 34.6 (1.5)  34.5 (1.5)  34.9 (1.9)  34.5 (1.9)  0.268 | 113  112  117  114 | 39.1 (1.6)  39.2 (1.6)  39.3 (2.1)  38.9 (2.3)  0.509 | 115  113  121  118 | 9.9 (0.6)  9.8 (0.7)  9.7 (1.2)  9.8 (0.9)  0.313 |
|  |  |  |  |  |  |  |  |  |  |  |  |
| Plasma-Se | 1  2  3  4  p | 117  116  119  122 | 3544.5 (606.1)  3573.7 (494.0)  3608. 0 (592.4)  3562.8 (624.1)  0.862 | 117  116  119  122 | 51.3 (2.4)  51.3 (2.6)  51.4 (3.0)  51.1 (3.6)  0.816 | 117  116  117  122 | 34.6 (1.6)  34.8 (1.4)  34.7 (2.0)  34.5 (1.9)  0.590 | 115  110  116  116 | 39.0 (1.8)  39.3 (1.4)  39.3 (2.2)  38.9 (2.2)  0.265 | 117  112  119  120 | 9.8 (1.0)  9.8 (0.9)  9.8 (0.9)  9.9 (0.7)  0.809 |
|  |  |  |  |  |  |  |  |  |  |  |  |
| Fe | 1  2  3  4  p | 122  114  116  121 | 3493.1 (625.2)  3625.8 (570.4)  3539.2 (553.6)  3637.1 (564.6)  0.163 | 122  114  116  121 | 50.9 (2.9)  51.4(3.3)  51.1 (3.0)  51.7 (2.4)  0.161 | 122  114  114  121 | 34.3 (1.7)  34.8 (1.7)  34.6 (2.0)  34.8 (1.5)  0.084 | 115  112  116  113 | 39.0 (2.0)  39.3 (2.0)  39.1 (2.1)  39.1 (1.6)  0.753 | 121  113  113  120 | 9.9 (0.6)  9.8 (0.8)  9.6 (1.4)  9.8 (0.4)  0.180 |
|  |  |  |  |  |  |  |  |  |  |  |  |
| Cu | 1  2  3  4  p | 103  108  126  123 | 3642.3 (642.0)  3675.1 (527.2)  3572.3 (579.0)  3439.9 (575.7)  **0.011** | 103  108  126  123 | 51.1 (4.1)  51.9 (2.4)  51.3 (2.3)  50.8 (2.7)  **0.042** | 103  108  124  123 | 34.7 (2.2)  34.9 (1.6)  34.7 (1.6)  34.4 (1.6)  0.173 | 100  107  121  118 | 39.3 (2.7)  39.4(1.5)  39.0 (1.6)  38.9 (1.7)  0.235 | 102  107  125  120 | 9.6 (1.5)  9.8 (0.6)  9.9 (0.7)  9.9 (0.5)  0.067 |
|  |  |  |  |  |  |  |  |  |  |  |  |
| Zn | 1  2  3  4  p | 121  120  112  120 | 3509.9 (656.3)  3574.0 (648.2)  3612.3 (504.5)  3599.9 (490.3)  0.533 | 121  120  112  120 | 51.0 (3.3)  51.1 (3.7)  51.5 (2.2)  51.6 (2.2)  0.266 | 119  120  112  120 | 34.5 (2.2)  34.6 (1.9)  34.6 (1.3)  34.8 (1.4)  0.640 | 117  115  110  114 | 38.8 (2.3)  39.0 (2.2)  39.3 (1.6)  39.4 (1.4)  0.127 | 119  118  110  120 | 9.7 (1.2)  9.8 (0.8)  9.9 (0.4)  9.8 (0.9)  0.380 |
|  |  |  |  |  |  |  |  |  |  |  |  |
| Mg | 1  2  3  4  p | 85  83  84  77 | 3501.5 (595.1)  3590.7 (642.9)  3499.3 (535.2)  3616.6 (496.8)  0.435 | 85  83  84  77 | 51.3 (2.6)  51.4 (3.8)  51.0 (2.1)  51.4 (2.3)  0.824 | 85  83  83  77 | 34.4 (1.4)  34.9 (2.1)  34.6 (1.4)  34.7 (1.4)  0.326 | 81  81  81  73 | 38.8 (1.7)  38.9 (2.3)  38.9 (1.6)  39.3 (1.4)  0.328 | 85  81  84  75 | 9.9 (0.5)  9.8 (0.8)  9.7 (1.2)  9.9 (0.5)  0.414 |
|  |  |  |  |  |  |  |  |  |  |  |  |
| Ca | 1  2  3  4  p | 81  75  85  88 | 3672.1 (512.9)  3534.0 (570.7)  3570.3 (544.4)  3432.9 (630.2)  0.055 | 81  75  85  88 | 51.7 (2.3)  51.2 (3.7)  51.3 (2.2)  50.8 (2.8)  0.207 | 81  75  85  87 | 34.9 (1.4)  34.6 (2.0)  34.5 (1.5)  34.5 (1.6)  0.327 | 79  71  84  82 | 39.3 (1.4)  38.9 (2.3)  39.0 (1.4)  38.7 (2.0)  0.125 | 80  74  84  87 | 9.9 (0.3)  9.5 (1.4)  9.9 (0.6)  9.9 (0.5)  **0.005** |

p-value calculated by One-way ANOVA on crude non-adjusted data
