# Supplementary material for: Pregnant Inuit Women’s Exposure to Metals and Association with Fetal Growth Outcomes: ACCEPT 2010–2015
Source: Int J Environ Res Public Health. 2019 Apr 1;16(7):1171. doi: 10.3390/ijerph16071171 (PMC6479494; doi:10.3390/ijerph16071171)
Supplement: Supplementary file 1 [file ijerph-16-01171-s001.zip › Table S3-S5. Metal Association Outcome.docx]

**Tabel S3**: Association between prenatal exposure to metals and ***birth length*** for both genders

|  | Raw data | | | Adjusted^1^ | | | | Adjusted^2^ | | | Adjusted^3^ | | |
| --- | --- | --- | --- | --- | --- | --- | --- | --- | --- | --- | --- | --- | --- |
| Metal (µg/L) | n | β (95%CI) | p | n | β (95%CI) | p | n | | β (95%CI) | p | n | β (95%CI) | p |
| Hg | 481 | -0.015 (-0.054;0.024) | 0.440 | 265 | -0.029 (-0.071;0.013) | 0.181 | 255 | | -0.011 (-0.049;0.027) | 0.559 | 265 | -0.026 (-0.068;0.016) | 0.223 |
| Pb | 481 | -0.021 (-0.059;0.017) | 0.273 | 265 | -0.005 (-0.050;0.041) | 0.841 | 255 | | 0.004 (-0.036;0.045) | 0.833 | 265 | -0.010 (-0.055;0.036) | 0.677 |
| As | 481 | -0.003 (-0.052;0.046) | 0.906 | 265 | 0.024 (-0.027;0.075) | 0.359 | 255 | | 0.011 (-0.037;0.059) | 0.645 | 265 | 0.026 (-0.025;0.077) | 0.318 |
| Cd | 481 | **-0.241 (-0.481;-0.001)** | **0.049** | 265 | **-0.308 (-0.556;-0.061)** | **0.015** | 255 | | **-0.228 (-0.450;-0.007)** | **0.043** | 265 | **-0.303 (-0.549;-0.057)** | **0.016** |
| Cr | 481 | -0.001 (-0.008;0.006) | 0.784 | 265 | 0.004 (-0.004;0.012) | 0.330 | 255 | | 0.0001 (-0.007;0.008) | 0.974 | 265 | 0.004 (-0.005;0.012) | 0.374 |
| Mn | 481 | 0.006 (-0.001;0.023) | 0.484 | 265 | 0.008 (-0.008;0.025) | 0.323 | 255 | | 0.002 (-0.013;0.017) | 0.787 | 265 | 0.007 (-0.009;0.023) | 0.395 |
| Ni | 481 | -6.67x10^-5^ (-0.001;0.001) | 0.842 | 265 | -0.0002 (-0.001;0.0004) | 0.574 | 255 | | 7.25x10^-6^ (-0.00047;0.00046) | 0.976 | 265 | -0.0001 (-0.001;0.0004) | 0.646 |
| Se | 481 | -0.0002 (-0.002;0.001) | 0.791 | 265 | 0.0002 (-0.0011;0.0014) | 0.800 | 255 | | 0.0003 (-0.001;0.001) | 0.573 | 265 | 0.0002 (-0.0010;0.0014) | 0.783 |
| P-Se | 481 | -0.004 (-0.014;0.006) | 0.381 | 265 | -0.004 (-0.014;0.006) | 0.401 | 255 | | -0.002 (-0.011;0.007) | 0.645 | 265 | -0.005 (-0.015;0.005) | 0.368 |
| Fe | 481 | 2.03x10^-6^ (-0.000001;0.000005) | 0.181 | 265 | 1.50x10^-7^ (-0.000003;0.000003) | 0.923 | 255 | | -2.04 x10^-7^ (-0.000003;0.000003) | 0.889 | 265 | -5.35x10^-7^ (-0.000003;0.000003) | 0.736 |
| Cu | 468 | -0.0007 (-0.001;-0.00002) | 0.099 | 265 | -0.001 (-0.001;0.0001) | 0.104 | 255 | | -0.0002 (-0.001;0.001) | 0.848 | 265 | -0.001 (-0.002;0.0001) | 0.100 |
| Zn | 481 | 0.0002(-0.00005;0.00047) | 0.115 | 265 | 6.70x10^-5^(-0.0002;0.0004) | 0.618 | 255 | | 9.13x10^-6^ (-0.00023;0.00026) | 0.941 | 265 | 2.15x10^-5^ (-0.0002; 0.0003) | 0.874 |
| Mg | 329 | 1.62x10^-5^ (-0.00004;0.00010) | 0.638 | 219 | -1.77x10^-5^ (-0.00009;0.00005) | 0.616 | 209 | | -2.87x10^-5^ (-0.0001;0.00004) | 0.392 | 219 | -2.72x10^-5^ (-0.000098;0.000044) | 0.452 |
| Ca | 329 | -2.23x10^-5^ (-0.000051;0.000006) | 0.096 | 219 | -2.10x10^-5^ (-0.00005;0.00001) | 0.115 | 209 | | -1.05x10^-5^ (-0.000036;0.000015) | 0.415 | 219 | -1.96x10^-5^ (-0.00005;0.000007) | 0.143 |

^1^:Core adjustment: Age, BMI, alcohol during pregnancy, cotinine, parity, n-3/n-6 ratio; ^2^:Core and gestation age adjustment; ^3^:Core and region adjustment

**Tabel S4**: Association between prenatal exposure to metals and ***head circumference*** for both genders

|  | Raw data | | | Adjusted^1^ | | | | Adjusted^2^ | | | Adjusted^3^ | | |
| --- | --- | --- | --- | --- | --- | --- | --- | --- | --- | --- | --- | --- | --- |
| Metal (µg/L) | n | β (95%CI) | p | n | β (95%CI) | p | n | | β (95%CI) | p | n | β (95%CI) | p |
| Hg | 479 | -0.023 (-0.046;-0.0003) | **0.047** | 264 | -0.019 (-0.047;0.008) | 0.163 | 254 | | -0.007 (-0.031;0.017) | 0.547 | 264 | -0.019 (-0.046;0.009) | 0.179 |
| Pb | 479 | -0.017 (-0.039;0.005) | 0.138 | 264 | -0.001 (-0.030;0.028) | 0.943 | 254 | | 0.002 (-0.021;0.030) | 0.715 | 264 | -0.002 (-0.032;0.027) | 0.873 |
| As | 479 | -0.020 (-0.050;0.009) | 0.169 | 264 | -0.005 (-0.038;0.028) | 0.769 | 254 | | -0.011 (-0.042;0.019) | 0.471 | 264 | -0.004 (-0.038;0.029) | 0.793 |
| Cd | 479 | -0.120 (-0.264;0.024) | 0.102 | 264 | -0.145 (-0.306;0.015) | 0.076 | 254 | | -0.077 (-0.219;0.064) | 0.282 | 264 | -0.144 (-0.305;0.017) | 0.079 |
| Cr | 479 | 0.001 (-0.005;0.003) | 0.795 | 264 | 0.001 (-0.005;0.006) | 0.762 | 254 | | -0.002 (-0.007;0.002) | 0.349 | 264 | 0.001 (-0.005;0.006) | 0.791 |
| Mn | 479 | 0.004 (-0.006;0.014) | 0.425 | 264 | 0.002 (-0.009;0.012) | 0.744 | 254 | | -0.003 (-0.012;0.007) | 0.545 | 264 | 0.001 (-0.009;0.012) | 0.788 |
| Ni | 479 | -0.0002 (-0.001;0.0002) | 0.314 | 264 | -0.0003 (-0.0006;0.0001) | 0.136 | 254 | | -0.0002 (-0.0005;0.0001) | 0.271 | 264 | -0.0003 (-0.001;0.00008) | 0.146 |
| Se | 479 | -0.001 (-0.001;0.0004) | 0.196 | 264 | -0.0002 (-0.001;0.00060) | 0.637 | 254 | | 9.38x10^-5^ (-0.001;0.001) | 0.784 | 264 | -0.00018 (-0.001;0.001) | 0.644 |
| P-Se | 479 | -0.004 (-0.010;0.002) | 0.176 | 264 | -0.004 (-0.011;0.002) | 0.194 | 254 | | -0.002 (-0.008;0.004) | 0.464 | 264 | -0.004 (-0.011;0.002) | 0.185 |
| Fe | 479 | 8.97x10^-7^ (-0.000001;0.000003) | 0.319 | 264 | -8.48x10^-7^ (-0.000003;0.000001) | 0.365 | 254 | | -1.39x10^-6^ (-0.000003;0.000001) | 0.133 | 264 | -1.08x10^-6^ (-0.000003;0.000001) | 0.296 |
| Cu | 466 | -0.000442 (-0.001;0.00002) | 0.063 | 264 | -0.0003 (-0.0007;0.0004) | 0.554 | 254 | | 0.0001 (-0.0003;0.0007) | 0.671 | 264 | -0.0002 (-0.0007;0.000359) | 0.364 |
| Zn | 479 | 7.08x10^-5^ (-0.00009;0.00023) | 0.348 | 264 | -9.24x10^-5^ (-0.00026;0.00008) | 0.286 | 254 | | **-0.00016 (-0.00031;-0.000006)** | **0.038** | 264 | -0.0001 (-0.000257;0.000087) | 0.221 |
| Mg | 328 | 1.39x10^-5^ (-0.00002;0.00007) | 0.491 | 219 | -5.68x10^-6^ (-0.000051;0.000038) | 0.802 | 209 | | -1.71x10^-5^ (-0.00006;0.00002) | 0.427 | 219 | -6.23x10^-6^ (-0.000053;0.000039) | 0.790 |
| Ca | 328 | -6.74x10^-6^ (-0.000025;0.000010) | 0.390 | 219 | 2.00x10^-6^ (-0.000015;0.000019) | 0.816 | 209 | | 9.64x10^-6^ (-0.000006;0.000026) | 0.243 | 219 | 2.07x10^-6^ (-0.000015;0.000019) | 0.811 |

^1^:Core adjustment: Age, BMI, alcohol during pregnancy, cotinine, parity, n-3/n-6 ratio; ^2^:Core and gestation age adjustment; ^3^:Core and region adjustment

**Tabel S5**: Association between prenatal exposure to metals and ***gestational age*** for both genders

|  | Raw data | | | Adjusted^1^ | | | Adjusted^2^ | | |
| --- | --- | --- | --- | --- | --- | --- | --- | --- | --- |
| Metal (µg/L) | n | β (95%CI) | p | n | β (95%CI) | p | n | β (95%CI) | p |
| Hg | 464 | -0.015 (-0.041;0.011) | 0.244 | 255 | -0.024(0.053;0.004) | 0.090 | 255 | -0.022 (-0.051;0.006) | 0.122 |
| Pb | 464 | -0.010 (-0.037;0.016) | 0.441 | 255 | -0.015 (-0.045;0.015) | 0.332 | 255 | -0.017 (-0.048;0.013) | 0.258 |
| As | 464 | -0.007 (-0.041;0.026) | 0.663 | 255 | 0.009 (-0.027;0.045) | 0.631 | 255 | 0.010 (-0.026;0.046) | 0.581 |
| Cd | 464 | -0.140 (-0.300;0.020) | 0.086 | 255 | -0.116 (-0.284;0.052) | 0.176 | 255 | -0.110 (-0.277;0.058) | 0.198 |
| Cr | 464 | -0.001 (-0.005;0.004) | 0.699 | 255 | 0.005 (-0.001;0.010) | 0.104 | 255 | 0.004 (-0.001;0.010) | 0.114 |
| Mn | 464 | 0.006 (-0.006;0.017) | 0.332 | 255 | 0.007 (-0.004;0.018) | 0.207 | 255 | 0.007 (-0.004;0.018) | 0.230 |
| Ni | 464 | -0.0001 (-0.001;0.0003) | 0.499 | 255 | -0.0002 (-0.001;0.0001) | 0.299 | 255 | -0.0002 (-0.0005;0.0002) | 0.340 |
| Se | 464 | -0.0003 (-0.001;0.001) | 0.532 | 255 | -0.0002 (-0.001;0.001) | 0.577 | 255 | -0.0002 (-0.001;0.001) | 0.597 |
| P-Se | 464 | -0.002 (-0.009;0.004) | 0.468 | 255 | -0.003 (-0.009;0.004) | 0.446 | 255 | -0.003 (-0.009;0.004) | 0.422 |
| Fe | 464 | 1.88x10^-6^ (-1.70x10^-7^;0.000004) | 0.072 | 255 | 4.77x10^-7^ (-0.000002;0.000003) | 0.667 | 255 | 1.48x10^-7^ (-0.000002;0.000002) | 0.895 |
| Cu | 454 | **-0.0006 (-0.0011;-0.00007)** | **0.026** | 255 | **-0.001 (-0.001;-0.00006)** | **0.048** | 255 | **-0.001 (-0.001;-0.00002)** | **0.042** |
| Zn | 464 | **0.0003 (0.0001;0.0005)** | **0.003** | 255 | 8.94x10^-5^ (-0.00009;0.000276) | 0.966 | 255 | 6.78x10^-5^ (-0.0001;0.00026) | 0.468 |
| Mg | 316 | **5.80x10^-5^** **(0.00001;0.00010)** | **0.011** | 209 | 1.40x10^-5^ (-0.00003;0.00006) | 0.574 | 209 | 6.28x10^-6^ (-0.00004;0.00006) | 0.804 |
| Ca | 316 | **-**2.62x10^-5^ **(-0.00004;-0.00001)** | **0.004** | 209 | -1.31x10^-5^ (-0.00003;0.00001) | 0.168 | 209 | -1.22x10^-5^ (-0.00003;0.000006) | 0.198 |

^1^:Core adjustment: Age, BMI, alcohol during pregnancy, cotinine, parity, n-3/n-6 ratio; ^2^:Core and region adjustment
