# Supplementary material for: Pregnant Inuit Women’s Exposure to Metals and Association with Fetal Growth Outcomes: ACCEPT 2010–2015
Source: Int J Environ Res Public Health. 2019 Apr 1;16(7):1171. doi: 10.3390/ijerph16071171 (PMC6479494; doi:10.3390/ijerph16071171)
Supplement: Supplementary file 1 [file ijerph-16-01171-s001.zip › Table S6-S13. Metal Association by Gender.docx]

**Table S6**: Association between prenatal exposure to metals and ***birth weight*** for female children

|  | Raw data | | | Adjusted^1^ | | | Adjusted^2^ | | | Adjusted^3^ | | |
| --- | --- | --- | --- | --- | --- | --- | --- | --- | --- | --- | --- | --- |
| Metal (µg/L) | n | β (95%CI) | p | n | β (95%CI) | p | n | β (95%CI) | p | n | β (95%CI) | p |
| Hg | 227 | -7.543 (-17.604;2.519) | 0.141 | 131 | -4.548 (-14.852;5.756) | 0.384 | 127 | 0.481 (-9.630;8.668) | 0.917 | 131 | -3.758 (-14.120;6.605) | 0.474 |
| Pb | 227 | -5.385 (-17.241;6.471) | 0.372 | 131 | **-14.355 (-27.235;-1.475)** | **0.029** | 127 | -10.713 (-22.16; 0.729) | 0.066 | 131 | **-15.200 (-28.055;-2.345)** | **0.021** |
| As | 227 | -2.222 (-14.392;9.948) | 0.719 | 131 | 6.144 (-7.679;19.967) | 0.381 | 127 | 6.961 (-6.104;20.026) | 0.294 | 131 | 7.656 (-6.252;21.563) | 0.278 |
| Cd | 227 | **-74.722 (-131.732;-17.713)** | **0.010** | 131 | -57.622 (-115.697;0.453) | 0.052 | 127 | -40.207 (-91.955;11.542) | 0.127 | 131 | -54.087 (-112.419;4.246) | 0.069 |
| Cr | 227 | 0.059 (-1.733;1.850) | 0.949 | 131 | **3.362 (0.122;6.603)** | **0.042** | 127 | 2.374 (-0.534;5.283) | 0.109 | 131 | **3.441 (0.212;6.670)** | **0.037** |
| Mn | 227 | 2.551 (-4.319;9.421) | 0.465 | 131 | 4.342 (-4.647;13.332) | 0.341 | 127 | 5.199 (-2.710;13.109) | 0.196 | 131 | 4.416 (-4.547;13.378) | 0.331 |
| Ni | 227 | -0.146 (-0.298;0.005) | 0.059 | 131 | **-0.175 (-0.306;-0.044)** | **0.009** | 127 | -0.094 (-0.214;0.025) | 0.122 | 131 | -0.168 (-0.300;-0.037) | **0.012** |
| Se | 227 | -0.024 (-0.308;0.259) | 0.866 | 131 | 0.072 (-0.199;0.342) | 0.601 | 127 | 0.107 (-0.129;0.343) | 0.370 | 131 | 0.077 (-0.192;0.347) | 0.572 |
| P-Se | 227 | -0.534 (-3.219;2.151) | 0.695 | 131 | -0.869 (-3.715;1.978) | 0.547 | 127 | -0.577 (-3.094;1.940) | 0.651 | 131 | -0.979 (-3.817;1.860) | 0.496 |
| Fe | 227 | 0.001 (-0.0001;0.002) | 0.075 | 131 | 0.0001 (-0.001; 0.001) | 0.849 | 127 | 0.0002 (-0.001;0.001) | 0.852 | 131 | 7.22x10^-5^ (-0.001;0.001) | 0.907 |
| Cu | 220 | -0.163 (-0.393;0.067) | 0.164 | 131 | **-0.300 (-0.553;0.048)** | **0.020** | 127 | -0.158 (-0.388;0.072) | 0.177 | 131 | **-0.321 (-0.573;-0.069)** | **0.013** |
| Zn | 227 | 0.043 (-0.037; 0.124) | 0.291 | 131 | 0.005 (-0.087;0.098) | 0.113 | 127 | 0.011 (-0.070;0.093) | 0.784 | 131 | -0.001 (-0.093;0.092) | 0.991 |
| Mg | 156 | **0.027 (0.005; 0.049)** | **0.018** | 109 | 0.002 (-0.022;0.026) | 0.861 | 105 | 0.003 (-0.018;0.024) | 0.787 | 109 | -0.0002 (-0.024;0.024) | 0.989 |
| Ca | 156 | **-0.012 (-0.022;-0.002)** | **0.022** | 109 | -0.010 (-0.020;0.001) | 0.085 | 105 | -0.006 (-0.016;0.003) | 0.183 | 111 | -0.010 (-0.020;0.001) | 0.083 |

p-value calculated by One-way ANOVA; ^1^: Core: Age. BMI. alcohol during pregnancy. cotinine. parity. n-3/n-6 ratio; ^2^: Core and gestation age; ^3^: Core and region

**Table S7**: Association between prenatal exposure to metals and ***birth length*** for female children

|  | Raw data | | | | Adjusted^1^ | | | | Adjusted^2^ | | | | Adjusted^3^ | | |  |
| --- | --- | --- | --- | --- | --- | --- | --- | --- | --- | --- | --- | --- | --- | --- | --- | --- |
| Metal (µg/L) | n | β (95%CI) | p | n | | β (95%CI) | p | n | | β (95%CI) | p | n | | β (95%CI) | p | |
| Hg | 227 | -0.035 (-0.094;0.023) | 0.156 | 131 | | -0.018 (-0.068;0.033) | 0.208 | 127 | | -0.003 (-0.041;0.035) | 0.506 | 131 | | -0.017 (-0.068;0.034) | 0.259 | |
| Pb | 227 | -0.023 (-0.087;0.042) | 0.665 | 131 | | -0.031 (-0.091;0.029) | 0.164 | 127 | | -0.029 (-0.074;0.01) | 0.324 | 131 | | -0.033 (-0.094;0.027) | 0.134 | |
| As | 227 | -0.005 (-0.076;0.066) | 0.781 | 131 | | -0.014 (-0.054;0.083) | 0.415 | 127 | | 0.017 (-0.038;0.073) | 0.493 | 131 | | 0.017 (-0.052;0.086) | 0.320 | |
| Cd | 227 | -0.238 (-0.569;0.093) | 0.139 | 131 | | -0.189 (-0.476;0.099) | 0.071 | 127 | | -0.118 (-0.336;0.100) | 0.1306 | 131 | | -0.202 (-0.435;0.032) | 0.090 | |
| Cr | 227 | 0.001 (-0.009;0.011) | 0.635 | 131 | | 0.012 (-0.005;0.028) | 0.248 | 127 | | 0.005 (-0.008;0.017) | 0.447 | 131 | | 0.012 (-0.004;0.028) | 0.232 | |
| Mn | 227 | 0.019 (-0.019;0.056) | 0.775 | 131 | | 0.028 (-0.016;0.072) | 0.342 | 127 | | 0.027 (-0.006;0.060) | 0.190 | 131 | | 0.029 (-0.015;0.073) | 0.334 | |
| Ni | 227 | -0.0002 (-0.0011;0.0007) | 0.596 | 131 | | -0.0003 (-0.0010;0.0003) | 0.205 | 127 | | 0.00007 (-0.00044;0.00057) | 0.847 | 131 | | -0.0003 (-0.0010;0.0004) | 0.243 | |
| Se | 227 | 0.0002 (-0.0015; 0.0018) | 0.996 | 131 | | 0.0005 (-0.0009;0.0018) | 0.477 | 127 | | 0.0006 (-0.0004;0.0016) | 0.293 | 131 | | 0.0005 (-0.0009;0.0018) | 0.455 | |
| P-Se | 227 | -0.003 (-0.018;0.013) | 0.427 | 131 | | 0.009 (-0.005;0.022) | 0.764 | 127 | | 0.005 (-0.005;0.016) | 0.568 | 131 | | 0.008 (-0.005;0.022) | 0.808 | |
| Fe | 227 | 0.000006 (-0.000001;0.000012) | 0.167 | 131 | | 0.000003 (-0.000003;0.000009) | 0.828 | 127 | | 0.000001 (-0.000003;0.000006) | 0.814 | 131 | | 0.000003 (-0.000003;0.000009) | 0.879 | |
| Cu | 220 | 0.00006 (-0.00122;0.00135) | 0.855 | 131 | | -0.0004 (-0.0016;0.0008) | 0.323 | 127 | | 0.0003 (-0.0006;0.0013) | 0.998 | 131 | | -0.0004 (-0.0016;0.0008) | 0.267 | |
| Zn | 227 | 0.0003 (-0.0001;0.0008) | 0.373 | 131 | | 0.0003 (-0.0001;0.0008) | 0.944 | 127 | | 0.0001 (-0.0002;0.0005) | 0.759 | 131 | | 0.0003 (-0.0001;0.0008) | 0.969 | |
| Mg | 156 | 0.0001 (0.00002;0.0002) | 0.108 | 109 | | 0.00004 (-0.00009;0.00016) | 0.878 | 105 | | -0.000002 (-0.000091;0.000095) | 0.926 | 109 | | 0.00003 (-0.00009;0.00015) | 0.763 | |
| Ca | 156 | -0.00003 (-0.00009;0.00003) | 0.250 | 109 | | -0.00002 (-0.00008;0.00003) | 0.181 | 105 | | -0.00002 (-0.00006;0.00003) | 0.325 | 109 | | -0.00002 (-0.00008;0.00003) | 0.180 | |

p-value calculated by One-way ANOVA; ^1^: Core: Age. BMI. alcohol during pregnancy. cotinine. parity. n-3/n-6 ratio; ^2^: Core and gestation age; ^3^: Core and region

**Table S8**: Association between prenatal exposure to metals and ***head circumference*** for female children

|  | Raw data | | | Adjusted^1^ | | | | Adjusted^2^ | | | Adjusted^3^ | | |
| --- | --- | --- | --- | --- | --- | --- | --- | --- | --- | --- | --- | --- | --- |
| Metal (µg/L) | n | β (95%CI) | p | | n | β (95%CI) | p | n | β (95%CI) | p | n | β (95%CI) | p |
| Hg | 225 | -0.029 (-0.061;0.004) | 0.088 | | 130 | -0.011 (-0.041;0.018) | 0.447 | 126 | 0.005 (-0.022;0.032) | 0.988 | 130 | -0.006 (-0.042;0.029) | 0.433 |
| Pb | 225 | -0.022 (-0.060;0.017) | 0.356 | | 130 | -0.022 (-0.064;0.020) | 0.142 | 126 | -0.099 (-0.051;0.13) | 0.357 | 130 | -0.022 (-0.064;0.021) | 0.146 |
| As | 225 | -0.027 (-0.069;0.015) | 0.104 | | 130 | -0.018 (-0.065;0.030) | 0.508 | 126 | 0.010 (-0.049;0.029) | 0.602 | 130 | 0.019 (-0.067;0.029) | 0.489 |
| Cd | 225 | -0.121 (-0.312;0.070) | 0.213 | | 130 | -0.120 (-0.320;0.079) | 0.126 | 126 | -0.061 (-0.215;0.092) | 0.303 | 130 | -0.126 (-0.328;0.076) | 0.121 |
| Cr | 225 | 0.0005 (-0.0057;0.0067) | 0.678 | | 130 | 0.006 (-0.005;0.017) | 0.481 | 126 | 0.001 (-0.007;0.010) | 0.742 | 130 | 0.006 (-0.006;0.017) | 0.486 |
| Mn | 225 | 0.013 (-0.010;0.036) | 0.545 | | 130 | 0.004 (-0.027;0.034) | 0.836 | 126 | 0.002 (-0.021;0.026) | 0.988 | 130 | 0.003 (-0.028;0.034) | 0.835 |
| Ni | 225 | -0.0003 (-0.0008;0.0002) | 0.213 | | 130 | **-0.0004 (-0.001;-0.00004)** | **0.032** | **126** | 0.0001 (-0.0005;0.0002) | 0.287 | 130 | -0.0004 (-0.001;0.00004) | **0.031** |
| Se | 225 | -0.0003 (-0.0013;0.0007) | 0.350 | | 130 | 0.00008 (-0.00101;0.00085) | 0.753 | 126 | 0.00002 (-0.00071;0.00070) | 0.893 | 130 | 0.00009 (-0.00102;0.00085) | 0.749 |
| P-Se | 225 | -0.001 (-0.011;0.008) | 0.400 | | 130 | 0.001 (-0.009;0.010) | 0.415 | 126 | -0.001 (-0.009;0.006) | 0.542 | 130 | 0.001 (-0.009;0.010) | 0.418 |
| Fe | 225 | 2.90x10^-6^ (-0.000000;0.000007) | 0.106 | | 130 | 0.000001 (-0.000003;0.000005) | 0.728 | 126 | -0.000001 (-0.000004;0.000002) | 0.568 | 130 | 0.000001 (-0.000003;0.000005) | 0.736 |
| Cu | 218 | -0.00006 (-0.00084;0.00071) | 0.658 | | 130 | -0.0006 (-0.0014;0.0003) | 0.150 | 126 | 0.0002 (-0.0008;0.0005) | 0.465 | 130 | -0.001 (-0.001;0.0003) | 0.155 |
| Zn | 225 | 0.00025 (-0.00003;0.00053) | 0.295 | | 130 | 0.00008 (-0.00023;0.00039) | 0.313 | 126 | -0.00006 (-0.00030;0.00018) | 0.327 | 130 | 0.00008 (-0.00023;0.00039) | 0.320 |
| Mg | 155 | 5.96x10^-5^ (-0.00008;0.00013) | 0.085 | | 109 | 0.00004 (-0.00004;0.00013) | 0.719 | 105 | -0.000009 (-0.000057;0.000075) | 0.827 | 109 | 0.00004 (-0.00004;0.00013) | 0.725 |
| Ca | 155 | -0.00002 (-0.00005;0.00002) | 0.244 | | 109 | 0.000004 (-0.000035;0.000043) | 0.887 | 105 | 0.000003 (-0.000028;0.000033) | 0.983 | 109 | -0.00004 (-0.00004;0.00004) | 0.888 |

p-value calculated by One-way ANOVA; ^1^: Core: Age. BMI. alcohol during pregnancy. cotinine. parity. n-3/n-6 ratio; ^2^: Core and gestation age; ^3^: Core and region

**Table S9**: Association between prenatal exposure to metals and ***gestation age*** for female children

|  | Raw | | | Adjusted^1^ | | | | Adjusted^2^ | | | |  |
| --- | --- | --- | --- | --- | --- | --- | --- | --- | --- | --- | --- | --- |
| Metal (µg/L) | n | β (95%CI) | p | | n | β (95%CI) | p | | n | β (95%CI) | p | |
| Hg | 221 | -0.026 (-0.069;0.017) | 0.112 | | 127 | -0.025 (-0.055;0.005) | 0.098 | | 127 | -0.023 (-0.052;0.007) | 0.136 | |
| Pb | 221 | 0.003 (-0.044;0.050) | 0.793 | | 127 | -0.024 (-0.062;0.014) | 0.208 | | 127 | -0.007 (-0.058;0.045) | 0.168 | |
| As | 221 | -0.012 (-0.066;0.042) | 0.475 | | 127 | 0.001 (-0.042;0.045) | 0.960 | | 127 | -0.007 (-0.070;0.056) | 0.799 | |
| Cd | 221 | -0.144 (-0.390;0.101) | 0.255 | | 127 | -0.108 (-0.280;0.063) | 0.213 | | 127 | -0.089 (-0.337;0.158) | 0.267 | |
| Cr | 221 | -0.001 (-0.008;0.007) | 0.433 | | 127 | 0.005 (-0.004;0.015) | 0.288 | | 127 | 0.010 (-0.004;0.024) | 0.269 | |
| Mn | 221 | -0.0003 (-0.0280;0.0274) | 0.551 | | 127 | -0.006 (-0.032;0.020) | 0.651 | | 127 | 0.004 (-0.033;0.042) | 0.657 | |
| Ni | 221 | -0.0004 (-0.0011;0.0003) | 0.158/ | | 127 | **-0.00049 (-0.001;-0.0001)** | **0.013** | | 127 | **-0.00046 (-0.001;-0.00008)** | **0.018** | |
| Se | 221 | -0.00014 (-0.00135;0.00107) | 0.561 | | 127 | -0.0002 (-0.0013;0.0010) | 0.655 | | 127 | -0.0001 (-0.0013;0.0010) | 0.690 | |
| P-Se | 221 | 0.001 (-0.010;0.012) | 0.521 | | 127 | 0.004 (-0.007;0.016) | 0.745 | | 127 | 0.004 (-0.008;0.015) | 0.681 | |
| Fe | 221 | 2.39x10^-6^ (-0.000000;0.000009) | 0.258 | | 127 | 6.24x10^-7^ (-0.000002;0.000008) | 0.729 | | 127 | 0.000003 (-0.000002;0.000008) | 0.785 | |
| Cu | 216 | -0.00017 (-0.00112;0.00078) | 0.866 | | 127 | -0.001 (-0.001;0.00006) | 0.073 | | 127 | -0.001 (-0.001;0.000003) | 0.051 | |
| Zn | 221 | 0.000168 (-0.0001;0.0005) | 0.288 | | 127 | 0.0002 (-0.0001;0.0006) | 0.723 | | 127 | 0.0002 (-0.0001;0.0006) | 0.621 | |
| Mg | 152 | **8.67x10^-5^(0.00003;0.00021)** | **0.026** | | 105 | 1.29x10^-5^ (-0.00005;0.00016) | 0.724 | | 105 | 0.00005 (-0.00006;0.00015) | 0.910 | |
| Ca | 152 | -0.00002 (-0.00006;0.00002) | 0.142 | | 105 | -9.06x10^-6^ (-0.000049;0.000046) | 0.592 | | 105 | 0.000002 (-0.000049;0.000046) | 0.580 | |

p-value calculated by One-way ANOVA; ^1^: Core: Age. BMI. alcohol during pregnancy. cotinine. parity. n-3/n-6 ratio; ^2^: Core and region

Male children

**Table S10**: Association between prenatal exposure to metals and ***birth weight*** for male children

|  | Raw data | | | Adjusted^1^ | | | Adjusted^2^ | | | | Adjusted^3^ | | | |  |
| --- | --- | --- | --- | --- | --- | --- | --- | --- | --- | --- | --- | --- | --- | --- | --- |
| Metal (µg/L) | n | β (95%CI) | p | n | β (95%CI) | p | | n | β (95%CI) | p | | n | β (95%CI) | p | |
| Hg | 253 | -1.273 (-13.091;10.545) | 0.832 | 134 | -1.830 (-24.640;20.979) | 0.874 | | 128 | 4.218 (-14.135;22.570) | 0.650 | | 134 | -2.287 (-25.079;20.504) | 0.843 | |
| Pb | 253 | -8.061 (-17.575;1.454) | 0.096 | 134 | 5.972 (-10.821;22.765) | 0.483 | | 128 | 5.727 (-7.427;18.881) | 0.390 | | 134 | 4.664 (-12.293;21.621) | 0.587 | |
| As | 253 | 8.948 (-6.974;24.870) | 0.269 | 134 | 12.718 (-8.295;33.730) | 0.233 | | 128 | 3.363 (-13.407;20.134) | 0.692 | | 134 | 11.707 (-9.381;32.794) | 0.274 | |
| Cd | 253 | -58.624 (-140.399;23.152) | 0.159 | 134 | -115.166 (-265.096; 34.764) | 0.131 | | 128 | -93.487 (-215.407;28.433) | 0.132 | | 134 | -136.834 (-288.800;15.133) | 0.077 | |
| Cr | 253 | 0.913 (-1.072;2.899) | 0.366 | 134 | 0.756 (-1.731;3.244) | 0.548 | | 128 | -0.682 (-2.642;1.278) | 0.492 | | 134 | 0.613 (-1.886;3.112) | 0.628 | |
| Mn | 253 | 0.700 (-3.187;4.587) | 0.723 | 134 | 0.962 (-3.568;5.491) | 0.675 | | 128 | -1.568 (-5.155;2.018) | 0.388 | | 134 | 0.681 (-3.871;5.233) | 0.768 | |
| Ni | 253 | 0.053 (-0.186;0.291) | 0.663 | 134 | 0.037 (-0.214;0.287) | 0.773 | | 128 | -0.058 (-0.250;0.135) | 0.553 | | 134 | 0.035 (-0.215;0.285) | 0.782 | |
| Se | 253 | 0.049 (-0.679;0.777) | 0.894 | 134 | 0.205 (-1.020;1.430) | 0.741 | | 128 | 0.436 (-0.527;1.398) | 0.372 | | 134 | 0.155 (-1.071;1.382) | 0.802 | |
| P-Se | 253 | 0.370 (-2.537;3.277) | 0.901 | 134 | -1.494 (-5.287;2.298) | 0.437 | | 128 | -0.602 (-3.628;2.424) | 0.694 | | 134 | -1.537 (-5.325;2.250) | 0.423 | |
| Fe | 253 | -1.12x10^-6^ (-0.001;0.001) | 0.998 | 134 | -3.43x10^-5^ (-0.001;0.001) | 0.942 | | 128 | -0.0002 (-0.001;0.001) | 0.550 | | 134 | -0.0003 (-0.001;0.001) | 0.622 | |
| Cu | 247 | **-0.262 (-0.471;-0.054)** | **0.014** | 134 | -0.164 (-0.452;0.124) | 0.263 | | 128 | -0.026 (-0.253;0.202) | 0.823 | | 134 | -0.149 (-0.438;0.141) | 0.311 | |
| Zn | 253 | 0.020 (-0.042;0.082) | 0.533 | 134 | 0.015 (-0.070;0.100) | 0.726 | | 128 | -0.032(-0.102;0.037) | 0.354 | | 134 | 0.002 (-0.085;0.090) | 0.957 | |
| Mg | 172 | -0.004 (-0.021;0.013) | 0.630 | 110 | 0.004 (-0.017;0.026) | 0.692 | | 104 | -0.002 (-0.021;0.016) | 0.797 | | 110 | 0.006 (-0.017;0.029) | 0.608 | |
| Ca | 172 | -0.002 (-0.009;0.004) | 0.435 | 110 | -0.004 (-0.012;0.003) | 0.272 | | 104 | -0.001 (-0.008;0.006) | 0.742 | | 110 | -0.005 (-0.013;0.003) | 0.235 | |

p-value calculated by One-way ANOVA; ^1^: Core: Age. BMI. alcohol during pregnancy. cotinine. parity. n-3/n-6 ratio; ^2^: Core and gestation age; ^3^: Core and region

**Table S11**: Association between prenatal exposure to metals and ***birth length*** for male children

|  | Raw data | | | | Adjusted^1^ | | | | Adjusted^2^ | | | | Adjusted^3^ | | |  |
| --- | --- | --- | --- | --- | --- | --- | --- | --- | --- | --- | --- | --- | --- | --- | --- | --- |
| Metal (µg/L) | n | β (95%CI) | p | n | | β (95%CI) | p | n | | β (95%CI) | p | n | | β (95%CI) | p | |
| Hg | 253 | 0.008 (-0.047;0.063) | 0.413 | 134 | | -0.012 (-0.112;0.088) | 0.719 | 128 | | 0.021 (-0.068;0.110) | 0.776 | 134 | | -0.020 (-0.120;0.079) | 0.079 | |
| Pb | 253 | -0.035 (-0.080;0.009) | 0.232 | 134 | | 0.016 (-0.058;0.090) | 0.479 | 128 | | 0.024 (-0.040;0.088) | 0.358 | 134 | | 0.020 (-0.055;0.095) | 0.591 | |
| As | 253 | 0.013 -0.058;0.085) | 0.554 | 134 | | 0.024 (-0.064;0.112) | 0.527 | 128 | | -0.003 (-0.080;0.074) | 0.999 | 134 | | 0.029 (-0.062;0.114) | 0.600 | |
| Cd | 253 | -0.218 (-0.582;0.145) | 0.462 | 134 | | -0.376 (-1.034; 0.281) | 0.259 | 128 | | -0.416 (-0.944;0.112) | 0.427 | 134 | | -0.472 (-1.138; 0.194) | 0.163 | |
| Cr | 253 | 0.002 (-0.007;0.011) | 0.592 | 134 | | 0.003 (-0.007;0.014) | 0.661 | 128 | | -0.001 (-0.011;0.008) | 0.721 | 134 | | 0.004 (-0.007;0.015) | 0.755 | |
| Mn | 253 | -0.001 (-0.019;0.017) | 0.701 | 134 | | 0.002 (-0.018;0.022) | 0.839 | 128 | | -0.006 (-0.023;0.012) | 0.511 | 134 | | 0.003 (-0.017;0.023) | 0.946 | |
| Ni | 253 | 0.0004 (-0.0008;0.0015) | 0.512 | 134 | | 0.0002 (-0.0009;0.0013) | 0.730 | 128 | | -0.0002 (-0.0011;0.0008) | 0.724 | 134 | | 0.0002 (-0.0009;0.0013) | 0.739 | |
| Se | 253 | 0.0002 (-0.0032;0.0036) | 0.792 | 134 | | 0.001 (-0.004;0.007) | 0.778 | 128 | | 0.002 (-0.002;0.007) | 0.425 | 134 | | 0.002 (-0.004;0.007) | 0.844 | |
| P-Se | 253 | -0.003 (-0.017;0.010) | 0.803 | 134 | | -0.011 (-0.027;0.006) | 0.239 | 128 | | -0.008 (-0.022;0.007) | 0.361 | 134 | | -0.011 (-0.027;0.006) | 0.228 | |
| Fe | 253 | 0.00000007 (-0.000003;0.000003) | 0.832 | 134 | | -0.000001 (-0.000005;0.000003) | 0.702 | 128 | | -0.000001 (-0.000005;0.000003) | 0.608 | 134 | | -0.000001 (-0.000005;0.000004) | 0.392 | |
| Cu | 247 | **-0.0014 (-0.0023;-0.0004)** | **0.037** | 134 | | -0.0008 (-0.002;0.00040) | 0.388 | 128 | | -0.0003 (-0.0014;0.0008) | 0.766 | 134 | | -0.0009 (-0.0021;0.0004) | 0.456 | |
| Zn | 253 | 0.00009 (-0.00020;0.00039) | 0.294 | 134 | | 0.00008 (-0.00030;0.00045) | 0.677 | 128 | | -0.00005 (-0.00038;0.00030) | 0.832 | 134 | | 0.0001 (-0.0003;0.0005) | 0.921 | |
| Mg | 172 | -0.00004 (-0.00012;0.00004) | 0.244 | 110 | | -0.00004 (-0.00013;0.00006) | 0.440 | 104 | | -0.00005 (-0.00015;0.00004) | 0.239 | 110 | | -0.00003 (-0.00014;0.00007) | 0.450 | |
| Ca | 172 | -0.00001 (-0.00004;0.00002) | 0.363 | 110 | | -0.000008 (-0.000042;0.000026) | 0.511 | 104 | | 0.000002 (-0.000035;0.000032) | 0.815 | 110 | | -0.00001 (-0.00005;0.00003) | 0.475 | |

p-value calculated by One-way ANOVA; ^1^: Core: Age. BMI. alcohol during pregnancy. cotinine. parity. n-3/n-6 ratio; ^2^: Core and gestation age; ^3^: Core and region

**Table S12**: Association between prenatal exposure to metals and ***head circumference*** for male children

|  | Raw data | | | | Adjusted^1^ | | | | Adjusted^2^ | | | Adjusted^3^ | | |  |
| --- | --- | --- | --- | --- | --- | --- | --- | --- | --- | --- | --- | --- | --- | --- | --- |
| Metal (µg/L) | n | β (95%CI) | p | n | | β (95%CI) | p | n | | β (95%CI) | p | n | β (95%CI) | p | |
| Hg | 253 | -0.023 (-0.059;0.013) | 0.367 | 134 | | -0.018 (-0.080;0.044) | 0.418 | 128 | | -0.007 (-0.062;0.048) | 0.565 | 134 | -0.017 (-0.079;0.046) | 0.396 | |
| Pb | 253 | -0.023 (-0.052;0.006) | 0.208 | 134 | | 0.021 (-0.024;0.067) | 0.265 | 128 | | 0.024 (-0.016;0.064) | 0.192 | 134 | 0.024 (-0.022;0.071) | 0.333 | |
| As | 253 | -0.0002 (-0.047;0.046) | 0.754 | 134 | | 0.009 (-0.046;0.064) | 0.604 | 128 | | -0.012 (-0.060;0.036) | 0.727 | 134 | 0.010 (-0.044;0.065) | 0.674 | |
| Cd | 253 | -0.068 (-0.291;0.154) | 0.546 | 134 | | -0.151 (-0.518;0.215) | 0.748 | 128 | | 0.015 (-0.314;0.344) | 0.814 | 134 | -0.129 (-0.502;0.244) | 0.589 | |
| Cr | 253 | 0.001 (-0.005;0.007) | 0.639 | 134 | | -0.0003 (-0.0070;0.0065) | 0.962 | 128 | | -0.004 (-0.010;0.002) | 0.225 | 134 | -0.00002 (-0.00678;0.00683) | 0.871 | |
| Mn | 253 | -0.002 (-0.014;0.010) | 0.746 | 134 | | 0.001 (-0.011;0.013) | 0.912 | 128 | | -0.004 (-0.015;0.007) | 0.362 | 134 | 0.002 (-0.011;0.014) | 0.992 | |
| Ni | 253 | 0.0001 (-0.0006;0.0008) | 0.791 | 134 | | 0.00005 (-0.00064;0.00073) | 0.904 | 128 | | -0.0002 (-0.0008;0.0004) | 0.498 | 134 | 0.00005 (-0.00064;0.00074) | 0.913 | |
| Se | 253 | -0.0004 (-0.0026;0.0018) | 0.903 | 134 | | 0.002 (-0.002;0.005) | 0.433 | 128 | | 0.0024 (-0.0005;0.0052) | 0.175 | 134 | 0.002 (-0.001;0.005) | 0.478 | |
| P-Se | 253 | -0.007 (-0.015;0.002) | 0.314 | 134 | | -0.005 (-0.016;0.005) | 0.387 | 128 | | -0.002 (-0.011;0.007) | 0.812 | 134 | -0.005 (-0.015;0.005) | 0.375 | |
| Fe | 253 | -0.000001 (-0.000003;0.000002) | 0.726 | 134 | | -0.000001 (-0.000004;0.000001) | 0.293 | 128 | | -0.000002 (-0.000004;0.000000) | 0.117 | 134 | -0.000001 (-0.000004;0.000001) | 0.129 | |
| Cu | 247 | -0.0005 (-0.001; 0.00008) | 0.089 | 134 | | 0.0001 (-0.0006;0.0009) | 0.692 | 128 | | 0.0005 (-0.0001;0.0012) | 0.130 | 134 | 0.00009 (-0.00070;0.00087) | 0.612 | |
| Zn | 253 | -0.00005 (-0.00024;0.00014) | 0.944 | 134 | | -0.00008 (-0.00031;0.00015) | 0.507 | 128 | | -0.000203 (-0.000410;0.000003) | 0.052 | 134 | -0.00006 (-0.00030;0.00018) | 0.325 | |
| Mg | 172 | -0.00002 (-0.00007;0.00004) | 0.426 | 110 | | -0.00003 (-0.00009;0.00003) | 0.372 | 104 | | -0.000041 (-0.000097;0.000014 | 0.143 | 110 | -0.00002 (-0.00009;0.00004) | 0.425 | |
| Ca | 172 | -0.0000004 (-0.000020;0.000019) | 0.856 | 110 | | 0.00001 (-0.00001;0.00003) | 0.390 | 104 | | 0.000018 (-0.000001; 0.000038 | 0.079 | 110 | 0.000009 (-0.000012;0.000030) | 0.433 | |

p-value calculated by One-way ANOVA; ^1^: Core: Age. BMI. alcohol during pregnancy. cotinine. parity. n-3/n-6 ratio; ^2^: Core and gestation age; ^3^: Core and region

**Table S13**: Association between prenatal exposure to metals and ***gestation age*** for male children

|  | Raw | | | Adjusted^1^ | | | Adjusted^2^ | | |
| --- | --- | --- | --- | --- | --- | --- | --- | --- | --- |
| Metal (µg/L) | n | β (95%CI) | p | n | β (95%CI) | p | n | β (95%CI) | p |
| Hg | 243 | -0.008 (-0.047;0.031) | 0.734 | 128 | -0.026 (-0.094;0.043) | 0.422 | 128 | -0.026 (-0.095;0.043) | 0.441 |
| Pb | 243 | -0.024 (-0.059;0.011) | 0.374 | 128 | -0.007 (-0.057;0.042) | 0.880 | 128 | -0.008 (-0.058;0.043) | 0.787 |
| As | 243 | 0.004 (-0.047;0.056 | 0.690 | 128 | 0.016 (-0.043;0.076) | 0.594 | 128 | 0.017 (-0.043;0.076) | 0.657 |
| Cd | 243 | -0.179 (-0.439;0.081) | 0.229 | 128 | -0.155 (-0.617;0.307) | 0.508 | 128 | -0.190 (-0.655;0.275) | 0.420 |
| Cr | 243 | 0.001 (-0.006;0.008) | 0.594 | 128 | 0.005 (-0.002;0.013) | 0.233 | 128 | 0.005 (-0.002;0.013) | 0.263 |
| Mn | 243 | 0.004 (-0.009;0.017) | 0.105 | 128 | 0.009 (-0.004;0.022) | 0.180 | 128 | 0.009 (-0.004;0.023) | 0.199 |
| Ni | 243 | 0.0005 (-0.0003;0.0013) | 0.177 | 128 | 0.0004 (-0.0003;0.0012) | 0.222 | 128 | 0.0004 (-0.0003;0.0012) | 0.228 |
| Se | 243 | -0.0002 (-0.0026;0.0022) | 0.864 | 128 | -0.002 (-0.005;0.002) | 0.264 | 128 | -0.002 (-0.002;0.002) | 0.244 |
| P-Se | 243 | -0.003 (-0.013;0.006) | 0.740 | 128 | -0.005 (-0.016;0.007) | 0.407 | 128 | -0.005 (-0.016;0.007) | 0.407 |
| Fe | 243 | 0.000001 (-0.000001;0.000004) | 0.159 | 128 | 1.810E-007 (-0.000003;0.000003) | 0.874 | 128 | 2.002E-007 (-0.000003;0.000003) | 0.879 |
| Cu | 238 | **-0.0012 (-0.0019;-0.0005)** | **0.002** | 128 | -0.0006 (-0.0014;0.0003) | 0.311 | 128 | -0.0006 (-0.0014;0.0003) | 0.344 |
| Zn | 243 | **0.0003 (0.0001;0.0005)** | **0.002** | 128 | 0.0002 (-0.0001;0.0004) | 0.168 | 128 | 0.0002 (-0.0001;0.0005) | 0.239 |
| Mg | 164 | 0.00004 (-0.00002;0.00011) | 0.173 | 104 | 0.00002 (-0.00005;0.00009) | 0.713 | 104 | 0.00001 (-0.00006;0.00009) | 0.798 |
| Ca | 164 | **-0.000027 (-0.000050;-0.000004)** | **0.012** | 104 | -0.00001 (-0.00004;0.00001) | 0.283 | 104 | -0.00001 (-0.00004;0.00001) | 0.309 |

p-value calculated by One-way ANOVA; ^1^: Core: Age. BMI. alcohol during pregnancy. cotinine. parity. n-3/n-6 ratio; ^2^: Core and region
